# Circulating Apolipoprotein L1 is associated with insulin resistance-induced abnormal lipid metabolism

**DOI:** 10.1038/s41598-019-51367-7

**Published:** 2019-10-16

**Authors:** Kenji Nishimura, Taichi Murakami, Toshihiro Sakurai, Masashi Miyoshi, Kiyoe Kurahashi, Seiji Kishi, Masanori Tamaki, Tatsuya Tominaga, Sumiko Yoshida, Kojiro Nagai, Hideharu Abe, Shu-Ping Hui, Kazuhiko Kotani, Toshio Doi

**Affiliations:** 1 Department of Nephrology, Graduate School of Biomedical Science, Tokushima University, Tokushima, Japan; 2 Faculty of Health Sciences, Hokkaido University, Sapporo, Japan; 3 Division of Medical Technology, Tokushima University Hospital, Tokushima, Japan; 4 Department of Hematology, Endocrinology and Metabolism, Graduate School of Biomedical Sciences, Tokushima University, Tokushima, Japan; 5 Department of Chronomedicine, Graduate School of Biomedical Science, Tokushima University, Tokushima, Japan; 6 Division of Community and Family Medicine, Center for Community Medicine, Jichi Medical University, Shimotsuke, Japan

**Keywords:** Dyslipidaemias, Dyslipidaemias, Translational research, Translational research

## Abstract

Circulating ApolipoproteinL1 (ApoL1) is a component of pre-β-high-density lipoprotein (HDL), however little is known about the relationship of ApoL1 with cardiometabolic factors. Considering previous studies reporting the correlation of ApoL1 to triglyceride, we have hypothesized that ApoL1 associates with insulin-related metabolism. The current study examined their associations in 126 non-diabetic subjects and 36 patients with type 2 diabetes (T2DM). Non-diabetic subjects demonstrated triglyceride (standardized coefficients [s.c.] = 0.204, *p* < 0.05), body mass index (s.c. =0.232, *p* < 0.05) and HDL cholesterol (s.c. = −0.203, *p* < 0.05) as independent determinant of ApoL1 levels, and the significant elevation of ApoL1 in metabolic syndrome. Lipoprotein fractionation analysis revealed the predominant distribution of ApoL1 in large HDL fraction, and the significant increase of ApoL1 in large LDL fraction in high ApoL1 samples with insulin resistance. In T2DM, ApoL1 was higher in T2DM with metabolic syndrome, however ApoL1 was lower with β cell dysfunction. Insulin significantly promotes ApoL1 synthesis and secretion in HepG2 cells. In conclusion, circulating ApoL1 may be associated with abnormal HDL metabolism in insulin resistant status. This may suggest a regulation of insulin signal on the ApoL1 level, leading to offer a novel insight to the ApoL1 biology.

## Introduction

Apolipoprotein L1 (ApoL1) was originally recognized as a component of human high-density lipoprotein (HDL) particles. ApoL1 possesses amphipathic helices, which confer a high level of lipophilicity; therefore, ApoL1 is entirely bound to HDL during the circulation of normolipidemic population^1^. APOL1 gene on chromosome 22 comprises putative sterol response elements in the promoter region^2^, through which the transcription of genes involved in lipid metabolism is presumed to be activated. Circulating ApoL1 levels correlate with the plasma triglyceride (TG) levels, and increases in subjects with a deficiency of cholesteryl ester transfer protein (CETP) that exchanges cholesterol and TG between the TG-rich lipoprotein and HDL^3^. Recent research reported that ApoL1-containing HDL subsets that comprise ApoA-I and ApoE are huge pre-β-HDL particles^4^. The evidence indicates that the HDL particles carrying ApoL1 are the characteristic subspecies in terms of HDL metabolism or bioactivity.

In innate immunity, HDL ApoL1 is the trypanolytic factor in human serum, which forms anion channels on the lysosomal membrane of *Trypanosoma brucei* that causes sleeping sickness^5,6^. Additionally, the association between plasma ApoL1 and the clinical characteristics including lipid-related factors in normolipidemic, dyslipidemic, or diabetic subjects, has also been explored. Duchateau *et al*. reported the independent positive correlation of ApoL1 with total cholesterol (Total-C) and TG in normolipidemia, and with TG in dyslipidemia. Moreover, it was observed that ApoL1 was elevated in the non-insulin dependent diabetic patients with hypertriglyceridemia^3^. Albert *et al*. reported that TG and hyperglycemia are the strongest predictors of plasma ApoL1 in dyslipidemic coronary artery disease subjects with low HDL cholesterol (HDL-C)^7^. Intriguingly, proteomic analysis demonstrated the positive correlation of the *in vitro* antioxidative capacity and ApoL1 peptide count on purified HDL determined by mass spectrometry^8^.

These data suggest that ApoL1 may be associated with lipid and glucose metabolisms or may exert an antioxidative effect as a distinct HDL subspecies in certain dyslipidemic subjects. We postulate that serum ApoL1 levels may elevate and exert certain biological activity in subjects with insulin resistance that frequently presents dyslipidemia marked by hypertriglyceridemia; however, the association between ApoL1 and insulin resistance has not been reported. The present study aimed to examine this association in the nondiabetic volunteers and patients with type 2 diabetes (T2DM) characterized by insulin resistance.

## Results

### Univariable linear regression analysis of characteristics relative to ApoL1 in non-diabetic volunteers

Initially, we examined the correlation of serum ApoL1 levels with clinical variables in all non-diabetic volunteers. In this study, 126 institute volunteers (male: 76 [60.3%], female: 50 [39.7%]) were enrolled, and their median age was 34.0 (26.3–41.0) years. Their median waist circumference (WC) and body mass index (BMI) were 79.0 (70.0–87.0) cm and 21.9 (19.5–24.9) kg/m^2^, and the mean systolic and diastolic blood pressure (BP) values were 113.8 ± 12.0 and 73.0 ± 11.0 mmHg, respectively. The percentage values of the habitual drinker and present smoker were 28.6% and 16.7%, respectively. The median value of serum ApoL1 was 24.0 (20.0–29.0) µg/mL. All characteristics in the serum and urine samples were within normal limits. Univariable linear regression analysis revealed the significant correlation of log ApoL1 with sex (standardized coefficients [s.c.] = 0.269, *p* < 0.01), log WC (s.c. = 0.412, *p* < 0.001), log BMI (s.c. = 0.430, *p* < 0.001), systolic BP (s.c. = 0.233, *p* < 0.01), diastolic BP (s.c. = 0.268, *p* < 0.01), low-density lipoprotein (LDL)-C (s.c. = 0.236, *p* < 0.01), log HDL-C (s.c. = −0.421, *p* < 0.001), log TG (s.c. = 0.416, *p* < 0.001), log γ-glutamyltransferase (γGTP) (s.c. = 0.342, *p* < 0.001), uric acid (s.c. = 0.343, *p* < 0.001), fasting blood sugar (FBS) (s.c. = 0.299, *p* < 0.001), log insulin (s.c. = 0.205, *p* < 0.05), and log adiponectin (s.c. = −0.362, *p* < 0.001) (Supplementary Table S1).

### Multivariable stepwise linear regression analysis of characteristics relative to ApoL1 in non-diabetic volunteers

Furthermore, we determined the independently correlated factors among these 13 characteristics found by univariable linear regression analysis. Multivariable stepwise linear regression analysis identified log BMI (s.c. = 0.232, *p* < 0.05), log TG (s.c. = 0.204, *p* < 0.05), and log HDL-C (s.c. = −0.203, *p* < 0.05) as the independent determinant of serum ApoL1 levels (Table 1). The positive correlation of log TG and log BMI and the inverse correlation of log HDL-C with log ApoL1 indicate the association of serum ApoL1 with typical features of dyslipidemia in obesity or metabolic syndrome (Mets).

**Table 1:** Multivariable stepwise linear regression analysis of characteristics relative to log ApoL1 in non-diabetic volunteers.

characteristic	B	standard deviation error	standarized coefficients	*p value*
**adjusted R**^**2**^**:0**.**251**				
log BMI (kg/m^2^)	0.396	0.165	0.232	<0.05
log TG (mg/dL)	0.127	0.058	0.204	<0.05
log HDL-C (mg/dL)	−0.265	0.127	−0.203	<0.05

BMI, TG and HDL-C were independent determinats of serum ApoL1 levels. BMI, body mass index;

TG, triglyceride; HDL-C, high-density lipoprotein cholesterol; ApoL1, apolipoproteinL1.

### The comparison between non-obesity, abdominal obesity, and pre-Mets/Mets

**Figure 1:**
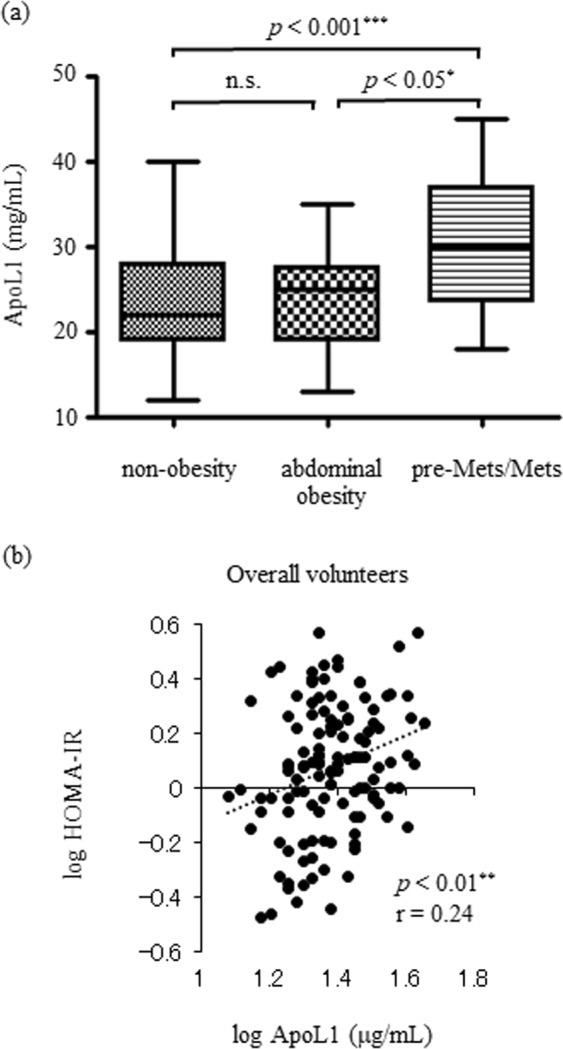
Serum ApoL1 levels in non-obesity, abdominal obesity and pre-Mets/Mets. (**a**) ApoL1 in pre-Mets/Mets is higher than non-obesity (p < 0.001) or abdominal obesity (p < 0.05). *P* value is calculated with *post hoc* comparison with Steel Dwass between non-obese, abdominal obese and pre-Mets/Mets. (**b**) Scatter plot of log ApoL1 relative to log HOMA-IR. *P* value is calculated with peason’s correlation coefficient test. Mets, metabolic syndrome; ApoL1, apolipoproteinL1; HOMA-IR, Homeostasis model assessment insulin resistance.

We classified 126 facility volunteers into 3 groups, namely, non-obese, abdominally obese, and preliminary metabolic syndrome (pre-Mets)/Mets, and compared the ApoL1 levels between these groups. No significant difference was observed in ApoL1, BMI, HDL-C, and TG between pre-Mets and Mets; hence, we combined both groups as pre-Mets/Mets. All data are listed in Table 2. The median ages of the non-obese, abdominally obese, and pre-Mets/Mets participants were 32.0 (26.0–38.0), 31.0 (27.3.0–41.0), and 41.5 (35.0–44.8) years, respectively, wherein the pre-Mets/Mets group is significantly older than the non-obese group (*p* < 0.01). The male percentage in the pre-Mets/Mets group was 81.8%, which was significantly higher than that of the non-obese group. In the non-obese group, the median WC and the mean BMI were 74.0 (68.0–80.8) cm and 20.7 ± 2.2 kg/m^2^ and all other characteristics were within normal limit. In the abdominally obese group defined as having central obesity without metabolic abnormality, the median WC and the mean BMI were 90.0 (87.0–96.3) cm and 25.5 ± 3.4 kg/m^2^, which were significantly higher than that of the non-obese group; however, all other characteristics revealed similar levels with the non-obese group. In the pre-Mets/Mets group, metabolic characteristics significantly increased compared to the non-obese group. Between the abdominally obese group and the pre-Mets/Mets group, characteristics except for the BP and fasting insulin revealed not significant difference, which was presumably due to lesser participants in these two groups or the large deviation. The median ApoL1 was 30.0 (24.8–35.8) µg/ml in the pre-Mets/Mets group, which was significantly higher than that in the non-obese (22.0 [19.0–27.8] µg/ml, *p* < 0.001) or abdominally obese group (25.0 [22.5–26.8] µg/ml, *p* < 0.05). No significant difference was observed in ApoL1 between the non-obese and abdominal obese groups (Fig. 1a). Insulin resistance, as a consequence of accumulating visceral fat, is the basic pathophysiology in Mets; therefore, we evaluated the relationship with Homeostasis model assessment insulin resistance (HOMA-IR) as insulin resistance index. As assumed, ApoL1 demonstrated the significant positive correlation with HOMA-IR (*p* < 0.01, r = 0.240; Fig. 1b). These data suggest that serum ApoL1 is associated with dyslipidemia in the Mets.

**Table 2:** Baseline characteristics of non-obesity, abdominal obesity and pre-Mets/Mets.

	non-obesity	abdominal obesity	pre-Mets/Mets	*p value*^a^	*p value*^b^		
					non-obesity vs abdominal obesity	non-obesity vs pre-Mets/Mets	abdominal obesity vs pre-Mets/Mets
N	90	14	22				
age (year)	32.0 (26.0–38.0)	31.0 (27.3–41.0)	41.5 (35.0–44.8)	<0.01**	n.s.	<0.01**	n.s.
male, n (%)	48 (53.3)	10 (71.4)	18 (81.8)	<0.05*	n.s.	<0.05*	n.s.
habitual drinker, n (%)	22 (24.4)	7 (50.0)	7 (31.8)	0.14	—	—	—
current smoker, n (%)	13 (14.4)	2 (14.3)	6 (27.3)	0.34	—	—	—
waist circumference (cm)	74.0 (68.0–80.8)	90.0 (87.0–96.3)	93.0 (90.0–97.5)	<0.001***	<0.001***	<0.001***	n.s.
BMI (kg/m2)	20.7 ± 2.2	25.5 ± 3.4	27.5 ± 2.9	<0.001***	<0.001***	<0.001***	n.s.
systolic BP (mmHg)	110.3 ± 10.1	115.6 ± 9.1	126.7 ± 11.9	<0.001***	n.s.	<0.001***	<0.01**
diastolic BP (mmHg)	70.7 ± 8.9	69.2 ± 10.2	84.7 ± 11.8	<0.001***	n.s.	<0.001***	<0.001***
Total-C (mg/dL)	189.4 ± 26.8	189.4 ± 26.8	206.2 ± 22.2	P < 0.05*	n.s.	<0.05*	n.s.
LDL-C (mg/dL)	104.0 ± 25.3	110.3 ± 22.5	131.4 ± 21.1	<0.001***	n.s.	<0.001***	n.s.
HDL-C (mg/dL)	67.1 ± 13.0	63.4 ± 14.0	53.4 ± 10.7	<0.001***	n.s.	<0.001***	n.s.
TG (mg/dL)	62.0 (51.3–86.5)	80.0 (55.5–95.8)	104.0 (35–142.8)	<0.01**	n.s.	<0.01**	n.s.
γGTP (U/L)	17.5 (13.8–23.3)	25.5 (16.3–36.5)	31.5 (19.0–65.8)	<0.001***	n.s.	<0.001***	n.s.
albumin (g/dL)	4.5 ± 0.3	4.4 ± 0.2	4.5 ± 0.3	0.52	—	—	—
uric acid (mg/dL)	5.0 ± 1.4	5.5 ± 1.6	5.9 ± 1.5	<0.05*	n.s.	<0.05*	n.s.
eGFR (ml/min)	90.2 ± 12.9	92.5 ± 14.9	85.2 ± 13.1	0.19	—	—	—
FBS (mg/dL)	92.4 ± 8.0	93.7 ± 4.3	101.7 ± 9.6	<0.001***	n.s.	<0.001***	n.s.
insulin (μU/mL)	4.9 (3.5–6.1)	5.3 (4.2–6.5)	7.6 (5.8–10.5)	<0.001***	n.s.	<0.001***	<0.05*
adiponectin (μg/mL)	11.8 ± 5.0	10.1 ± 6.3	7.0 ± 2.4	<0.001***	n.s.	<0.001***	n.s.
U-Alb/Cr (mg/gCr)	5.4 (4.5–7.6)	5.3 (4.0–6.2)	6.4 (4.0–9.6)	0.63	—	—	—
U-8OHdG/Cr(mg/gCr)	13.9 (10.9–17.7)	12.1 (9.2–15.4)	16.2 (11.6–20.1)	0.29	—	—	—
ApoL1 (μg/mL)	22.0 (19.0–27.8)	25.0 (22.5–26.8)	30.0 (24.8–35.8)	<0.001***	n.s.	<0.001***	<0.05*

Data are presented as mean ± standard deviation, as number (%) or as median (interquartile range) if skewed. ^a^Calculated with one way ANOVA in parametric characteristics or with Kruskal-Wallis in non-parametric characteristics. ^b^Calculated with Tukey in parametric characteristics or with Steel-Dwass in non-parametric characteristics between non-obese participants, abdominal obese participants and preMets/Mets. Mets, metabolic syndrome; BMI, body mass index; BP, blood pressure; Total-C, total cholesterol; LDL-C, low density lipoprotein cholesterol; HDL-C, high density lipoprotein cholesterol; TG, triglyceride; γ-GTP. γ-glutamyltransferase; eGFR, estimated glomerular filtration rate; FBS, fasting blood sugar; U-Alb/Cr, urine albumin to creatinine ratio; U-8OHdG/Cr, urine 8-Hydroxy-2′-Deoxyguanosine to creatinine ratio; ApoL1, apolipoproteinL1.

### Lipoprotein fractionation analysis in low and high ApoL1 subjects

**Figure 2:**
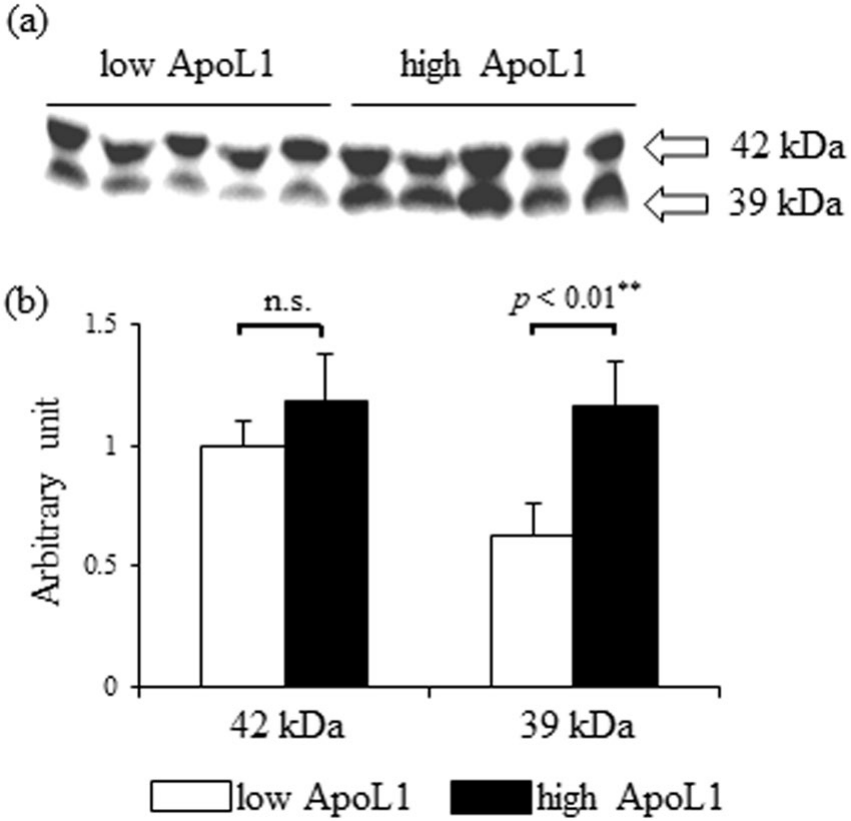
Immunoblot analysis of serum ApoL1 in low and high ApoL1 groups. (**a**) Western blotting detected 42 kDa and 39 kDa ApoL1 in serum. (**b**) Signal intensity of 39 kDa ApoL1 was significantly increased in high ApoL1 group. ApoL1, apolipoproteinL1.

Next, we compared ApoL1 distribution among lipoprotein fractions between low ApoL1 subjects (N = 5) and high ApoL1 subjects (N = 5). Serum ApoL1 levels were 24.2 ± 3.3 and 42.6 ± 9.9 µg/mL in low and high ApoL1 subjects, respectively. In high ApoL1 group, the WC (*p* < 0.05), BMI (*p* < 0.01), TG (*p* < 0.05), and insulin level (*p* < 0.05) were higher, whereas HDL-C (*p* < 0.05) and adiponectin (*p* < 0.01) levels were lower (Supplementary Table S2). Western blotting of serum in denatured condition identified two different signals of ApoL1, 42 kDa and 39 kDa (Fig. 2a), wherein not 42 kDa, but 39 kDa ApoL1 significantly increased in the high ApoL1 group (Fig. 2b).

**Figure 3:**
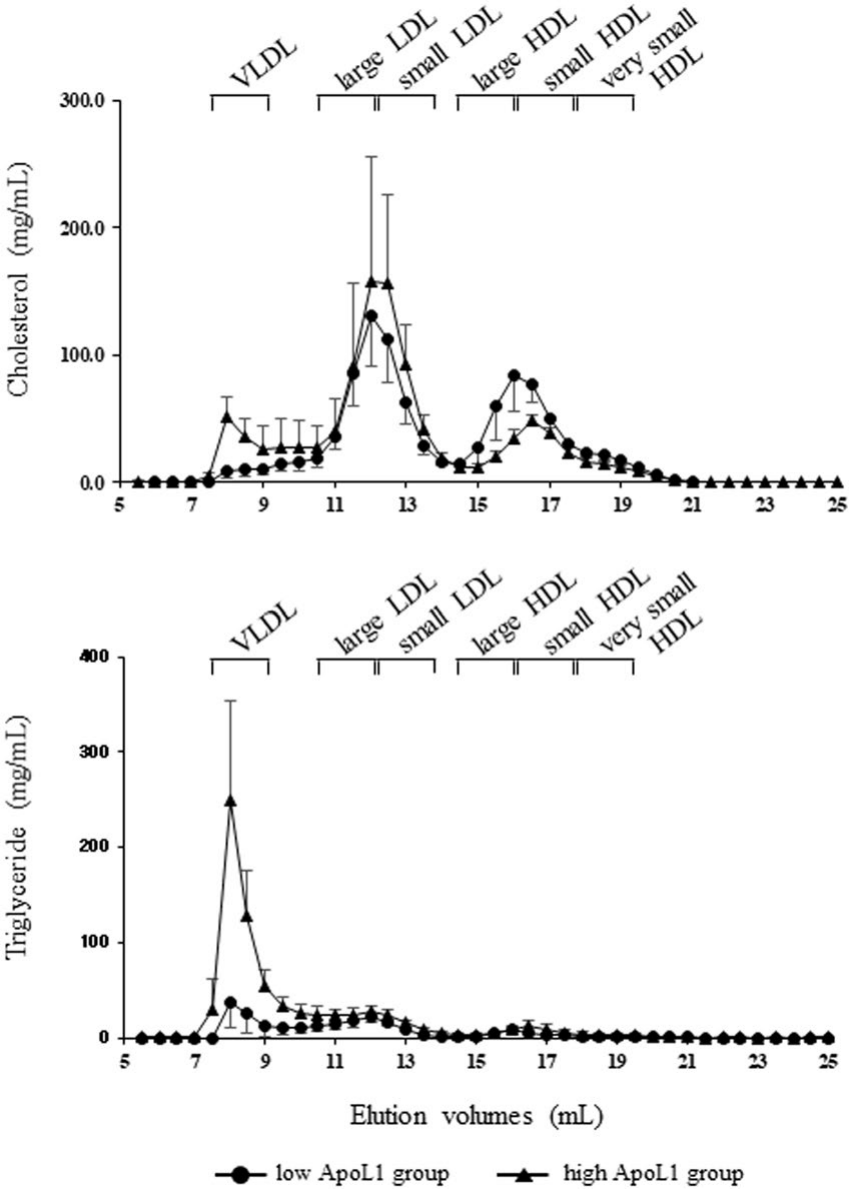
Lipid profile in lipoprotein fractionation analysis by gel filtration chromatography in low and high ApoL1 groups. VLDL, very low-density lipoprotein; LDL, low-density lipoprotein; HDL, high-density lipoprotein.

**Figure 4:**
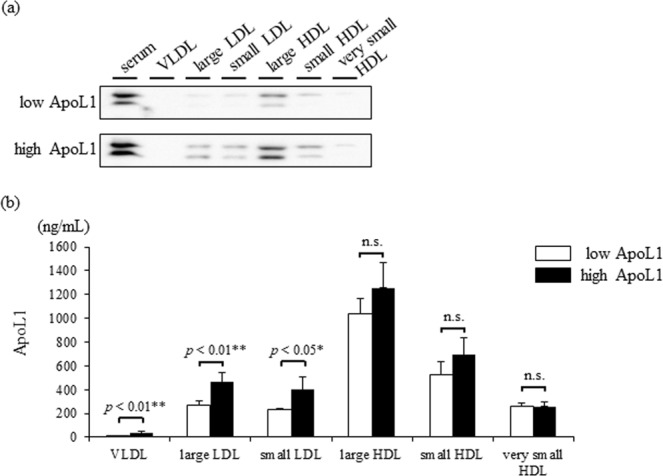
Alteration of ApoL1 distribution among lipoprotein fractions in low and high ApoL1 groups. (**a**) ApoL1 expression in lipoprotein fractions was determined by Western blotting. A representative. data of low ApoL1 group and high ApoL1 group are presented. (**b**) Quantitative evaluation of ApoL1 in lipoprotein fractions. *P* value was calculated with student’s *t*-test. VLDL, very low-density lipoprotein; LDL, low-density lipoprotein; HDL, high-density lipoprotein; ApoL1, apolipoproteinL1.

Furthermore, we quantified cholesterol, TG, and ApoL1 in lipoprotein fractions separated by gel filtration chromatography. The lipid profile is shown in Fig. 3 and Supplementary Table S3. Western blotting of ApoL1 in lipoprotein fractions revealed that ApoL1 was predominantly distributed in the large HDL fractions irrespective of the serum ApoL1 levels (Fig. 4a). ApoL1 is known to express in two different sizes of HDL complexes in large LDL and large HDL fractions in the normolipidemic subjects. In addition, ApoL1 expression levels are significantly enriched in the smaller complex^4^. Our data of western blotting is consistent with the results of previous studies. Interestingly, ApoL1 appeared to a lesser extent in LDL fractions of high ApoL1 sample. Quantification of ApoL1 by ELISA revealed the significant increase in the LDL fractions. ApoL1 in HDL fractions also indicated the trend to increase (Fig. 4b); however, it was non-significant. These findings indicate that two different size ApoL1-containing complexes may be differentially metabolized in abnormal HDL metabolism.

### Evaluation of circulating ApoL1 in T2DM

We examined ApoL1 expression in 36 patients with T2DM characterized by insulin resistance (Table 3). The mean age was 47.0 ± 9.9 years, and male frequency was 47.2%. The average of the abdominal circumference was 99.8 ± 18.2 cm, and BMI was 29.6 ± 7.9 kg/m^2^. The median FBS was 138.0 (123.5–186.0) mg/dL and the mean HbA1c was 9.7 ± 2.5%, thus indicating poor glycemic control. The median urine albumin-to-creatinine ratio was 29.1 (7.2–284.3) mg/gCr, in which overt albuminuria is 25.0%, microalbuminuria is 22.2%. ApoL1 revealed positive correlation with WC (*p* < 0.05, r = 0.38) and log TG (*p* < 0.05, r = 0.37), and negative correlation with log adiponectin (*p* < 0.05, r = 0.41) in overall patients with T2DM (Supplementary Fig. S1). However, unexpectedly, no significant difference of serum ApoL1 levels was observed between all T2DM patients and 90 nondiabetic non-obese volunteers. Therefore, we compared T2DM without Mets and with Mets (Table 3). ApoL1 was significantly lower in T2DM without Mets, wherein c-peptide also presented the significant decrease despite of hyperglycemia, thus indicating β cell dysfunction in this group. In addition, Interestingly, U-8OHdG/Cr was significantly lower in T2DM with Mets. Furthermore, ApoL1 is inversely correlated with log U-8OHdG/Cr (*p* < 0.05, r = 0.40).

**Table 3:** Baseline characteristics of patients with T2DM.

N	all	Mets (−)	Mets (+)	*p value*
	36	8	28	
age (year)	47.0 ± 9.9	52.5 ± 5.8	45.4 ± 10.3	0.07
male, n (%)	17 (47.2)	6 (75.0)	11 (39.3)	0.13
habitual drinker, n (%)	8 (18.6)	2 (25.0)	5 (17.9)	0.94
current smoker, n (%)	11 (30.6)	5 (62.5)	5 (17.9)	0.94
insulin administration, n (%)	7 (19.4)	1 (12.5)	6 (21.4)	0.7
waist circumference (cm)	99.8 ± 18.2	85.4 ± 12.1	103.9 ± 17.8	<0.01**
BMI (kg/m2)	29.6 ± 7.9	23.6 ± 2.8	31.3 ± 8.1	<0.05*
systolic BP (mmHg)	122.5 ± 17.5	116.1 ± 16.8	124.1 ± 17.6	0.29
diastolic BP (mmHg)	71.8 ± 8.9	64.7 ± 4.2	73.6 ± 8.9	<0.05*
Total-C (mg/dL)	230.9 ± 53.3	222.2 ± 38.7	233.3 ± 57.2	0.61
LDL-C (mg/dL)	133.1 ± 41.5	126.8 ± 43.4	134.4 ± 41.8	0.43
HDL-C (mg/dL)	47.6 ± 18.0	62.4 ± 23.5	43.4 ± 13.9	<0.01**
TG (mg/dL)	148.0 (111.3–235.3)	72.5 (109.3–60.3)	176.0 (131.8–362.8)	<0.01**
γGTP (U/L)	33.0 (19.0–47.5)	29.5 (14.5–52.5)	33.0 (22.8–44.8)	0.73
albumin (g/dL)	3.9 ± 0.5	3.9 ± 0.4	4.0 ± 0.5	0.59
uric acid (mg/dL)	5.8 ± 1.9	5.1 ± 2.2	6.0 ± 1.7	0.22
eGFR (ml/min)	83.0 ± 26.4	78.5 ± 19.6	84.3 ± 28.2	0.59
FBS (mg/dL)	138.0 (123.5–186.0)	151.0 (122.0–189.8)	138.0 (125.5–185.0)	0.98
C-peptide (ng/mL)	2.0 ± 1.3	1.3 ± 0.8	2.3 ± 1.3	<0.05*
HbA1c (%)	9.7 ± 2.5	10.3 ± 3.4	9.6 ± 2.3	0.52
adiponectin (μg/mL)	6.8 (5.1–10.7)	8.9 (6.8–12.1)	6.6 (5.0–9.3)	0.14
U-Alb/Cr (mg/gCr)	29.1 (7.2–284.3)	6.4 (3.4–38.7)	35.8 (10.8–409.5)	<0.05*
U-8OHdG/Cr (mg/gCr)	17.1 (11.6–22.7)	26.0 (20.8–28.7)	15.3 (9.3–20.1)	<0.01**
ApoL1 (μg/mL)	25.1 ± 8.7	18.4 ± 4.0	26.9 ± 8.9	<0.05*

Data are presented as mean ± standard deviation, as number (%) or as median (interquartile range) if skewed. P value was calculated with student’s t-test in parametric variables or Mann -Whitney’s U test was used in non-parametric variables for comparison between T2DM without Mets and T2DM with Mets. T2DM, type2 diabetes mellitus; Mets, metabolic syndrome; BMI, body mass index; BP, blood pressure; Total-C, total cholesterol; LDL-C, low-density lipoprotein cholesterol; HDL-C, highdensity lipoprotein cholesterol; TG, triglyceride; γGTP. γ-glutamyltransferase; eGFR, estimated glomerular filtration rate; FBS, fasting blood sugar; U-Alb/Cr, urine albumin to creatinine ratio; U-8OHdG/Cr, urine 8-Hydroxy-2’-Deoxyguanosine to creatinine ratio; ApoL1, apolipoproteinL1.

### *In vitro* examination on the insulin signaling-mediated ApoL1 expression and secretion in HepG2 cells

**Figure 5:**
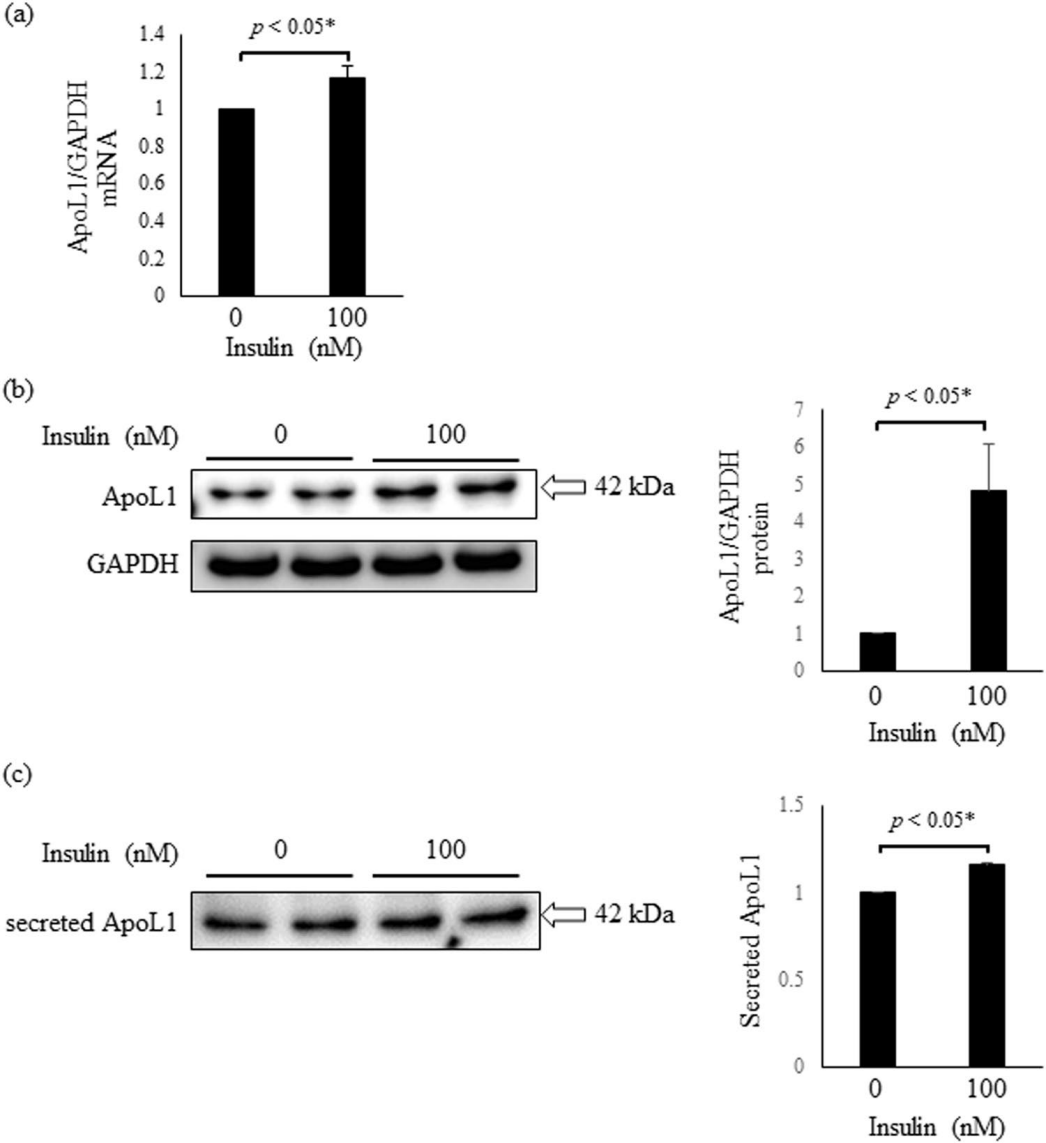
*in vitro* examination on insulin-mediated ApoL1 expression and secretion in HepG2 cells. (**a**) Expression of ApoL1 mRNA in HepG2 cells is determined by quantitative PCR. Arbitrary unit of ApoL1/GAPDH mRNA expression is shown. (**b**) ApoL1 protein expression in HepG2 cells is evaluated by western blotting. A representative data of ApoL1 in the absence of insulin or in the presence of 100 nM insulin are presented. Arbitrary unit of ApoL1/GAPDH protein expression is shown. (**c**) Secreted ApoL1 in culture media is evaluated by wester blotting. A representative data of ApoL1 in the absence of insulin or in the presence of 100 nM insulin are presented. Arbitrary unit of ApoL1 protein expression corrected by protein concentration in media is shown. *P* value was calculated with student’s *t*-test. Data is shown as mean SD of three to five independent experiments. GAPDH, glyceralhyde-3-phosphate dehydrogenase; ApoL1, apolipoproteinL1.

We hypothesized that insulin signal may regulate the ApoL1 expression in hepatic cells based on our clinical data, thus we examined the effect of insulin on ApoL1 expression in HepG2 cells and secretion in media. Previous studies reported HepG2 cells expresses insulin receptor and present insulin-dependent apoprotein secretion^9,10^. We incubated HepG2 cells in the absence of insulin or in the presence of 100 nM insulin. Incubation with 100 nM insulin for 6 h results in the significant upregulation of ApoL1 mRNA (*p* < 0.05) (Fig. 5a). ApoL1 protein levels in HepG2 cells were significantly increased after 12 h insulin stimulation (*p* < 0.05) (Fig. 5b). In addition, ApoL1 protein secretion in media showed the significant increase in the presence of 100 nM insulin for 24 h (*p* < 0.05) (Fig. 5c). ApoL1 protein in cells or meida was detected as a single band of 42 kDa by western blot, which may attribute to the some difference of proteolytic regulation between *in vivo* and *in vitro*. These data suggest that insulin signaling may regulate ApoL1 synthesis and secretion in hepatic cells

## Discussion

This observational study revealed several novel findings regarding insulin resistance-mediated ApoL1 biology in Mets and T2DM, including the elevation of serum ApoL1 in Mets. In nondiabetic volunteers, high BMI, hypertriglyceridemia, and low HDL-C were identified as independent characteristics that determined serum ApoL1 levels and are typical features associated with Mets. To the best of our knowledge, this is the first study to report a correlation between ApoL1 and HDL-C or BMI^3,7,11^. Specifically, serum ApoL1 in Mets was significantly higher than in non-obesity or abdominal obesity without insulin resistance. The abnormal lipid metabolism in Mets is characterized by elevated VLDL, hypertriglyceridemia, high levels of LDL-C, increased CETP activity, and low levels of HDL-C, in which insulin resistance and increased circulating free fatty acid (FFA) resulting from adipose tissue hyperplasia and hypertrophy are considered as fundamental pathogenesis^12–15^. This study also found a significant correlation between serum ApoL1 and HOMA-IR in all non-diabetic volunteers. These findings suggest that serum ApoL1 may not increase in obesity alone, but it may be elevated in non-diabetic Mets marked by insulin resistance-mediated dyslipidemia.

The ApoL1 increase in T2DM^3^ is consistent with the results of this study because insulin resistance is the common basal pathophysiology of Mets and T2DM. However, patients with T2DM unexpectedly demonstrated no significant increase in serum ApoL1 compared with the non-obese volunteers in this study. In contrast, those with T2DM exhibited a significant increase in WC, BMI, TG, and adiponectin compared with non-obese volunteers, thus suggesting a difference in ApoL1 regulation between Mets and T2DM. Considering that insulin secretory capacity in T2DM varies, we examined the presence of Mets in T2DM. T2DM without Mets had significantly lower c-peptide and ApoL1 levels, despite high FBS levels similar to T2DM with Mets, thereby indicating that absolute insulin deficiency due to β cell dysfunction is associated with low ApoL1 levels in T2DM without Mets. ApoL1 protein levels in the circulation are primarily determined by liver secretion^16^. We postulated that insulin, not hyperglycemia, promoted ApoL1 secretion in the Mets liver. We finally showed that insulin regulated ApoL1 synthesis and secretion in HepG2 cells. Hence, we conclude that ApoL1 synthesis and secretion in the liver attribute, in part, to insulin signaling in Mets or T2DM.

Alteration of the lipid and lipoprotein profile induced by human African trypanosomiasis (HAT), wherein ApoL1-containing particles function as lytic factors, has been reported. Huet *et al*. reported that patients with HAT presented with an altered lipid and lipoprotein profile, including hypertriglyceridemia, high LDL-C, low HDL-C, and reduced ApoA-1^17^. Lamour *et al*. found hypertriglyceridemia and lowered plasma levels of phospholipid and sphingolipid as metabolic markers in HAT^18^. Vervet monkeys infected with *Trypanosoma brucei* presented with hypoglycemia, hypertriglyceridemia, and low HDL-C^19^. Further, Trypanosoma infection-induced TNF has been shown to inhibit lipoprotein lipase activity in a rodent model^20^; therefore, increased lipolysis from adipose tissue may result in lipid abnormalities. These findings indicate some similarities in the pathophysiology of dyslipidemia between Mets and HAT. In addition, circulating ApoL1 expression was reported to be increased by *Trypanosoma brucei* infection^21^. The interaction of the altered lipid profile and increased ApoL1 expression remains unclear; however, some metabolic factors or pro-inflammatory cytokines induced by trypanosoma infection may alter lipid and lipoprotein metabolism in HAT.

A second major finding was the distinct expression and distribution pattern of ApoL1 in subjects with increased ApoL1 levels resulting from insulin resistance. Circulating ApoL1 was reported to show a bimodal distribution among lipoprotein fractions. Duchateau *et al*. reported that ApoL1 existed in the large HDL and VLDL fractions under nondenatured conditions, and that ApoL1-containing lipoproteins exhibited pre-β mobility^1^. Weckerle *et al*. also found ApoL1 in the LDL and large HDL fractions by FPLC separation, in which the ApoL1 protein was highly enriched in the large HDL particle size. They also found that these ApoL1-carrying HDL subspecies expressed ApoA-I and ApoE and migrated in the β/pre-β position^4^. Interestingly, plasma levels of pre-β HDL have been reported to increase in parallel with TG and BMI in Mets^22–24^. Lipoprotein fractionation analysis in this study indicated the predominant expression of ApoL1 in the large HDL fraction, independent of ApoL1 levels. However, ApoL1 in the LDL fraction, which corresponds with the larger ApoL1-containing complex, presented a significant increase (1.7-fold) in the high ApoL1 subjects, thus suggesting that two different size ApoL1-enriched complexes may be differentially metabolized in the abnormal HDL metabolic cycle of Mets.

Moreover, we detected serum ApoL1 at two different sizes of 42 and 39 kDa by western blotting, which are regarded as a precursor and a signal peptide-deleted activated form, respectively^1^. Compared with low ApoL1 subjects, high ApoL1 subjects exhibited a significant increase in total ApoL1 and 39 kDa ApoL1, thus indicating that circulating ApoL1 in Mets may be transcriptionally increased and proteolytically activated. The alteration in HDL apoprotein expression levels or distribution contributes to HDL metabolism or bioactivity in pathological conditions, including Mets^14,25–29^. These data suggest the possibility that the change in distribution or proteolytic activation of ApoL1 may be associated with ApoL1-containing HDL activity or metabolism in Mets.

The function of circulating APOL1 is another important point to consider. Therefore, we explored the association of ApoL1 levels with arterial stiffness and oxidative stress in T2DM. ApoL1 did not correlate with arterial stiffness, as evaluated by pulse wave velocity in this study, which may be consistent with the previous observation that ApoL1 levels did not correlate with the progression of coronary artery disease^7^. These data support that ApoL1 is not a strong proatherogenic molecule, although serum ApoL1 levels have been shown to be associated with known proatherogenic markers such as hypertriglyceridemia or low HDL-C.

Additionally, we measured urinary 8OHdG to evaluate oxidative DNA damage throughout the body^30^. U-8OHdG/Cr in T2DM without Mets was significantly higher than in T2DM with Mets, and log U-8OHdG/Cr was inversely correlated with serum ApoL1 levels. One of the possible explanations for this result is the antioxidative activity of ApoL1-enriched HDL. Little is known about the anti-oxidative capacity of ApoL1. Davidson *et al*. reported a positive correlation of ApoL1 peptide count on HDL by mass spectrometry and the anti-LDL oxidative capacity of HDL. In that study, the correlational network analysis found a strong relationship between ApoL1, paraoxonase1, paraoxonase2, and ApoF, thus suggesting potent anti-oxidative activity of these apoproteins^8^. Another possibility is that the oxidative stress-mediated β cell dysfunction may result in reduced insulin secretion^31^ and ApoL1 production in T2DM without Mets. A third possible explanation is the association between urinary 8OHdG and ApoL1 in kidney cells. ApoL1 protein is expressed in glomerular podocytes or proximal tubular cells, and the expression levels have been shown to decrease in diseased kidney. Conversely, urinary 8OHdG excretion increases in human and rodent kidney diseases^32–34^. ApoL1 in kidney cells is assumed to be derived from both endogenous synthesis and extracellular resource, and to serve a functional role^35,36^. ApoL1 localized in kidney cells may affect urinary 8OHdG excretion. Our data suggest several hypotheses on the ApoL1 effect; however, further investigation is required to clarify the causal association and mechanism between ApoL1 and oxidative stress.

This study had certain limitations. First, this study was conducted with our institute volunteers, in which the job types were slightly biased; therefore, the baseline data may differ from the general population. However, the pre-Mets/Mets ratio in our subjects (total 17.4%, 11.1% aged 20–40 years, 33.3% aged 20–40 years) did not differ from recent data of the Japan National Health and Nutrition Survey. Thus, the correlation between ApoL1 and clinical factors in this study is similar to that in the Japanese population with insulin resistance. Another limitation is that we did not examine gene mutations related to kidney disease in black Americans. The gene variant is known to determine trypanolytic activity or prognosis of kidney diseases^6,37^. Proteomic analysis revealed variant-related quantitative changes in apoproteins on ApoL1-containing HDL subspecies^4^; however, it remains unclear if these variants alter lipid metabolism, HDL function, or cardiovascular disease susceptibility^38–40^. Furthermore, the gene variants have not been examined in the Japanese population. Therefore, our study may provide insight into variant-independent ApoL1 biology.

In summary, the current observational study and *in vitro* examination demonstrated insulin resistance-mediated ApoL1 synthesis and secretion in the liver and alteration of ApoL1 distribution between lipoprotein fractions in Mets. These data may provide new insights into ApoL1 biology in insulin resistance-mediated abnormal lipid metabolism.

## Material and Method

### Design and subjects

This study enrolled 126 non-diabetic volunteers from age 20 to age 60. These non-diabetic volunteers who consisted of doctor, nurse, clinical laboratory technician, medical representative and medical student were recruited from our institute with their consent. We measured BP, height, body weight and WC, and collected serum and urine after an overnight fasting. According to the criteria of International Diabetes Federation, Mets was defined as having more than any two of the four factors including raised TG ($\geqq$150 mg/dL), reduced HDL-C (<40 mg/dL in males or <50 mg/dL in females), raised BP (systolic BP $\geqq$ 130 or diastolic BP $\geqq$ 85 mmHg), or raised FBS ($\geqq$100 mg/dL), in addition to central obesity (WC: male $\geqq$ 90 cm and female $\geqq$ 80 cm). Subject with abdominal obesity and one of the four factors was classified as pre-Mets. Subject with central obesity and without metabolic abnormality was defined as abdominal obesity. In addition, 36 patients with T2DM from age 20 to age 60 who were hospitalized in Tokushima University Hospital for the purpose of glycemic control were also registered as diabetic participants with their consent. For the analysis of ApoL1 distribution among lipoprotein fractions in low ApoL1 subjects and high ApoL1 subjects, we collected additional serum samples from 5 males in the top 25th percentile and the bottom 25th percentile of serum ApoL1 levels, respectively.

### Blood collection, biochemical analysis, and sample

Venous blood and urine were collected after an overnight fasting. Serum was then isolated by centrifugation (2,500 g for 10 min) and stored at −80 °C until analysis. Serum levels of Total-C, LDL-C, HDL-C, TG, γGTP, albumin, uric acid, creatinine, blood sugar, insulin, HbA1c and adiponectin, and urine levels of albumin, creatinine and 8OHdG were measured. 8OHdG was evaluated by ICR-001 (Techno Medica, Japan) and U-8OHdG/Cr was used as a marker of oxidative DNA damage. Estimated glomerular filtration rate of each participant was calculated from serum creatinine and age by using the 3-variable Japanese equation as follows; estimated glomerular filtration rate (ml/min/1.73 m^2^) = 194 X Age^−0.287^ X Creatinine (mg/dL)^−1.094^ (X 0.739 if female). BMI was calculated as weight in kilograms divided by height in meters squared. HOMA-IR, insulin resistance index, was calculated from FBS and insulin as follows; HOMA-IR = fasting insulin (μU/mL) X FBS (mg/dL)/405.

### Gel filtration chromatography

Serum samples (0.3 mL) were fractionated by gel filtration choromatography on a Superose 6 column (GE Healthcare, Little Chalfont, UK) in a liquid chromatography apparatus (Shimadzu, Kyoto, Japan) using a procedure modified from a previous report^41^. The column was eluted with 50 mmol/L phosphate buffer (pH7.4) containing 150 mmol/L NaCl and 1 mmol/L EDTA at a flow rate of 0.5 mL/min. Eluted fractions (0.5 mL each) were subjected to lipid analysis and immunoassays. Lipoprotein fractions were defined by eluted fraction volume as follows; very low-density lipoprotein (VLDL) 7.5–9 mL, large LDL 10.5–12 mL, small LDL 12–13.5 mL, large HDL 16–17.5 mL, small HDL, and very small HDL 17.5–19.0 mL.

### ApoL1 ELISA examination

ApoL1 concentration in serum and in lipoprotein fraction was measured by a human specific sandwich ELISA (KE00047) (proteintech, IL, USA). Briefly, serum was diluted 1:10,000 and assayed in duplicate according to the manufacture’s instructions. As for the examination of lipoprotein fractions, samples were diluted 1:250.

### Cell culture

HepG2 cells were cultivated in DMEM containing 10% fetal calf serum, 5%CO2 at 37 °C. For RNA isolation or protein extraction, HepG2 cells were seeded on 6-well plates at a density of 1 × 10^6^ cells/well and cultivated for 24 h. The culture medium was replaced with a fresh one without fetal calf serum, and cells were additionally incubated for 12 h before insulin administration. Cells were incubated in the absence of insulin or in the presence of 100 nM insulin (Wako, Tokyo, Japan) RNA or whole cell extract was collected after 6 h or after 12 h incubation with insulin, respectively. Culture media for 24 h insulin incubation was collected and concentrated by concentration column (Sartorius, Gottingen, Germany) for the evaluation of ApoL1 secretion.

### Western blotting

One μL of serum, 15 μg of whole cell lysate, 16 μL of concentrated culture media of HepG2 or 10 μL of lipoprotein solution were heated to 70 °C for 15 minutes in SDS gel-loading buffer, and applied to SDS gel electrophoresis, thereafter proteins were transferred to nitrocellulose filters (GE Healthcare, Little Chalfont, UK). The blots were incubated with rabbit anti-ApoL1 antibody (SIGMA, MO, USA) or rabbit anti-human GAPDH antibody (Abcam, Cambridge, UK), followed by incubation with horseradish peroxidase-conjugated goat anti-rabbit IgG (IBL, Gunma, Japan). Signal intensity of ApoL1 was measured by Image J.

### RNA isolation and quantitative PCR

The total RNA was extracted from cultured HepG2 cells by RNAiso Plus (Takara, Ootsu, Japan). Quantitative PCR was performed on BIO-RAD MJ Mini Personal Thermal Cycler (BIO-RAD, CA, USA) using SYBR Green PCR Master Mix (Applied Biosystems). The following PCR primers were used; human ApoL1: sense 5′- tgagattcaaaagccacactg-3′, antisense 5′- cgaggggcttactttgagga-3′ and human GAPDH: sense 5′-tttggctacagcaacagg-3′, antisense 5′-ggtctctctcttcctcttg-3′.

### Ethical approval

This study was approved by the Ethics Committee of Tokushima University Hospital (No.2128, No3306) and Hokkaido University (No.17–53–1), and was performed in compliance with the Helsinki Declaration. Written informed consent was obtained from all participants.

### Statistics

The results are shown as mean ± standard deviation, as number (%) or as median (interquartile range) if skewed. A test for normality was performed using the Kolmogorov-Smirnov test. Student’s *t*-test was used in parametric variables and Mann -Whitney’s U test was used in non-parametric variables for comparison between two groups. For the regression analysis, correlations were determined by univariate linear regression analysis, and multiple stepwise regression was used to confirm significant independent correlation. When data were non-normal distribution, log transformation was applied to correct skewness. For the assessment of potential differences in mean value, one-factor ANOVA was performed. Subsequently, as for the characteristic that presents significant difference between three groups, multiple comparison analysis was performed. Those that were normally distributed were subjected to multiple comparison by Tukey’s test, and those that were non-normal distribution were subjected to multiple comparison by Kruskal-Wallis test. Statistical analyses were performed with IBM SPSS statics. A *p* value $<$ 0.05 was considered significant.

## Supplementary information

Supplementary Table S1, Supplementary Table S2, Supplementary Table S3 and Supplementary Figure S1
