# Author Correction: Circulating Apolipoprotein L1 is associated with insulin resistance-induced abnormal lipid metabolism

**DOI:** 10.1038/s41598-019-55751-1

**Published:** 2019-12-27

**Authors:** Kenji Nishimura, Taichi Murakami, Toshihiro Sakurai, Masashi Miyoshi, Kiyoe Kurahashi, Seiji Kishi, Masanori Tamaki, Tatsuya Tominaga, Sumiko Yoshida, Kojiro Nagai, Hideharu Abe, Shu-Ping Hui, Kazuhiko Kotani, Toshio Doi

**Affiliations:** 1 Department of Nephrology, Graduate School of Biomedical Science, Tokushima University, Tokushima, Japan; 2 Faculty of Health Sciences, Hokkaido University, Sapporo, Japan; 3 Division of Medical Technology, Tokushima University Hospital, Tokushima, Japan; 4 Department of Hematology, Endocrinology and Metabolism, Graduate School of Biomedical Sciences, Tokushima University, Tokushima, Japan; 5 Department of Chronomedicine, Graduate School of Biomedical Science, Tokushima University, Tokushima, Japan; 6 Division of Community and Family Medicine, Center for Community Medicine, Jichi Medical University, Shimotsuke, Japan

Correction to: *Scientific Reports* 10.1038/s41598-019-51367-7, published online 16 October 2019

The original version of this Article incorrectly included Tatsuya Tominaga as a corresponding author. For correspondence and requests for materials, please contact Taichi Murakami (c-tamurakami@eph.pref.ehime.jp). This error has now been corrected in the PDF and HTML versions of this Article. The Supplementary Information file was correct at the time of publication.

