# Supplementary material for: Circulating Apolipoprotein L1 is associated with insulin resistance-induced abnormal lipid metabolism
Source: Sci Rep. 2019 Oct 16;9:14869. doi: 10.1038/s41598-019-51367-7 (PMC6795879; doi:10.1038/s41598-019-51367-7)
Supplement: Supplementary file 1 — Supplementary Table S1, Supplementary Table S2, Supplementary Table S3 and Supplementary Figure S1 [file 41598_2019_51367_MOESM1_ESM.pdf]

Circulating Apolipoprotein L1 is associated with insulin resistance-induced abnormal lipid metabolism

Kenji Nishimura[1)], Taichi Murakami[1)], Toshihiro Sakurai[2)], Masashi Miyoshi[3)], Kiyoe Kurahashi[4)], Seiji Kishi[1)], Masanori Tamaki[1)], Tatsuya Tominaga[5)], Sumiko Yoshida[4)], Kojiro Nagai[1)], Hideharu Abe[1)], Shu-Ping Hui[2)], Kazuhiko Kotani[6)], Toshio Doi[1)]

1) Department of Nephrology, Graduate School of Biomedical Science, Tokushima University, Tokushima, Japan.
2) Faculty of Health Sciences, Hokkaido University, Sapporo, Japan
3) Division of Medical Technology, Tokushima University Hospital, Tokushima, Japan.
4) Department of Hematology, Endocrinology and Metabolism, Graduate School of Biomedical Sciences, Tokushima University, Tokushima, Japan.
5) Department of Chronomedicine, Graduate School of Biomedical Science, Tokushima University, Tokushima, Japan.
6) Division of Community and Family Medicine, Center for Community Medicine, Jichi Medical University, Shimotsuke, Japan.

Corresponding author
Taichi Murakami
Department of Nephrology, Tokushima University Graduate School of Biomedical Science
3-18-15Kuramoto, Tokushima, Tokushima 770-8503 Japan
Tell:+81-88-633-7184, Fax:+81-88-633-9245,
E-mail: c-tamurakami@eph.pref.ehime.jp, tatsunoya@gmail.com

Supplementary Table S1 Univariable linear regression analysis of characteristics relative to log ApoL1 in non-diabetic volunteers

characteristic	B	standard deviation error	standarized coefficients	*p* value
log age (year)	-0.022	0.095	-0.021	0.819
male	0.066	0.021	0.269	< 0.01**
habitual drinker	0.035	0.024	0.131	0.144
current smoker	0.030	0.029	0.094	0.298
log waist circumference (㎝)	0.805	0.160	0.412	< 0.001***
log BMI (kg/m^2)	0.752	0.142	0.430	< 0.001***
systolic BP (mmHg)	0.002	0.001	0.233	< 0.01**
diastolic BP (mmHg)	0.003	0.001	0.268	< 0.01**
Total-C (mg/dL)	0.001	0.001	0.104	0.248
LDL-C (mg/dL)	0.001	0.001	0.236	< 0.01**
log HDL-C (mg/dL)	-0.556	0.108	-0.421	< 0.001***
log TG (mg/dL)	0.262	0.051	0.416	< 0.001***
log γGTP (U/L)	0.136	0.033	0.342	< 0.001***
log albumin (g/dL)	0.575	0.425	0.121	0.178
uric acid（mg/dL）	0.028	0.007	0.343	< 0.001***
eGFR (ml/min)	0.001	0.001	-0.023	0.799
FBS (mg/dL)	0.004	0.001	0.299	< 0.001***
log insulin (μU/mL)	0.105	0.046	0.205	< 0.05*
log adiponectin (μg/mL)	-0.216	0.050	-0.362	< 0.001***
log U-Alb/Cr (mg/gCr)	0.024	0.033	0.066	0.463
log U-8OHdG/Cr (mg/gCr)	-0.053	0.062	-0.077	0.399

ApoL1, apolipoproteinL1**;** BMI, body mass index; BP, blood pressure; Total-C, total cholesterol; LDL-C, low-density lipoprotein cholesterol; HDL-C, high-density lipoprotein cholesterol; TG, triglyceride; γGTP. γ-glutamyltransferase; eGFR, estimated glomerular filtration rate; FBS, fasting blood sugar; U-Alb/Cr, urine albumin to creatinine ratio; U-8OHdG/Cr, urine 8-Hydroxy-2'-Deoxyguanosine to creatinine ratio**.**

Supplementary Table S2 Baseline characteristics of low and high ApoL1 groups

	low ApoL1	high ApoL1	*p value*
N	5	5	
age (year)	38.8±7.7	35.0±5.0	n.s.
male, n (%)	5 (100.0)	5 (100.0)	n.s.
waist circumference (㎝)	76.2±7.8	90.4±9.6	< 0.05*
BMI (kg/m^2)	20.6±1.1	26.2±3.3	< 0.01**
Systolic BP (mmHg)	118.0±6.0	114.4±3.2	n.s.
Diastolic BP (mmHg)	74.2±5.0	76.0±8.4	n.s.
Total-C (mg/dL)	187.0±10.8	218.4±92.8	n.s.
LDL-C (mg/dL)	97.4±27.0	145.2±93.5	n.s.
HDL-C (mg/dL)	76.0±17.9	46.8±8.3	< 0.05*
TG (mg/dL)	58.0±24.3	175.2±66.3	< 0.05*
γGTP (U/L)	28.8±12.4	64.6±44.8	n.s.
albumin (g/dL)	4.6±0.3	4.6±0.3	n.s.
uric acid（mg/dL）	6.3±0.5	6.4±1.8	n.s.
eGFR (ml/min)	83.7±8.4	93.3±11.0	n.s.
FBS (mg/dL)	97.4±6.7	106.4±15.9	n.s.
insulin (μU/mL)	3.1 (2.3-3.4)	10.6 (6.6-24.3)	< 0.05*
adiponectin (μg/mL)	11.0±1.0	6.1±1.0	< 0.01**
ApoL1 (μg/mL)	24.2±3.3	42.6±9.9	< 0.01**

Data are presented as mean ± standard deviation, as number (%) or as median (interquartile range) if skewed. *P* value was calculated with student’s *t*-test in parametric variables or Mann -Whitney's U test was used in non-parametric variables. BMI, body mass index; BP, blood pressure; Total-C, total cholesterol; LDL-C, low-density lipoprotein cholesterol; HDL-C, high-density lipoprotein cholesterol; TG, triglyceride; γGTP. γ-glutamyltransferase; eGFR, estimated glomerular filtration rate; FBS, fasting blood sugar; ApoL1, apolipoproteinL1**.**

Supplementary Table S3 Cholesterol and triglyceride levels in lipoprotein fractions of low and high ApoL1 groups

	Cholesterol (mg/mL)			Triglyceride (mg/mL)		
	low ApoL1	high ApoL1	*p* value	low ApoL1	high ApoL1	*p* value
VLDL	29.1±15.9	112.2±47.2	< 0.01**	77.5±59.8	432.5±165.2	< 0.01**
large LDL	252.8±74.7	288.7±189.3	n.s.	53.9±18.3	75.8±20.2	n.s.
small LDL	204.9±57.6	290.4±113.5	n.s.	29.6±6.6	48.9±11.3	< 0.05*
large HDL	171.6±67.5	66.4±13.2	< 0.05*	16.0±10.2	17.3±6.6	n.s.
small HDL	157.7±24.2	111.2±11.9	< 0.01**	12.2±4.3	27.9±12.9	< 0.05*
very small HDL	63.0±9.7	41.5±5.7	< 0.01**	6.0±4.7	11.0±5.3	n.s.

Data are presented as mean ± standard deviation. *P* value was calculated with student's *t*-test. VLDL-C, VLDL-TG, small LDL-TG and small HDL-TG were significantly higher, and large HDL-C, small HDL-C, very small HDL-C were significantly lower in high ApoL1 group. VLDL, very low-density lipoprotein; LDL, low-density lipoprotein; HDL, high-density lipoprotein; C, cholesterol; TG, triglyceride.

Supplementary Figure S1 Correlation of serum ApoL1 levels and clinical characteristics in T2DM

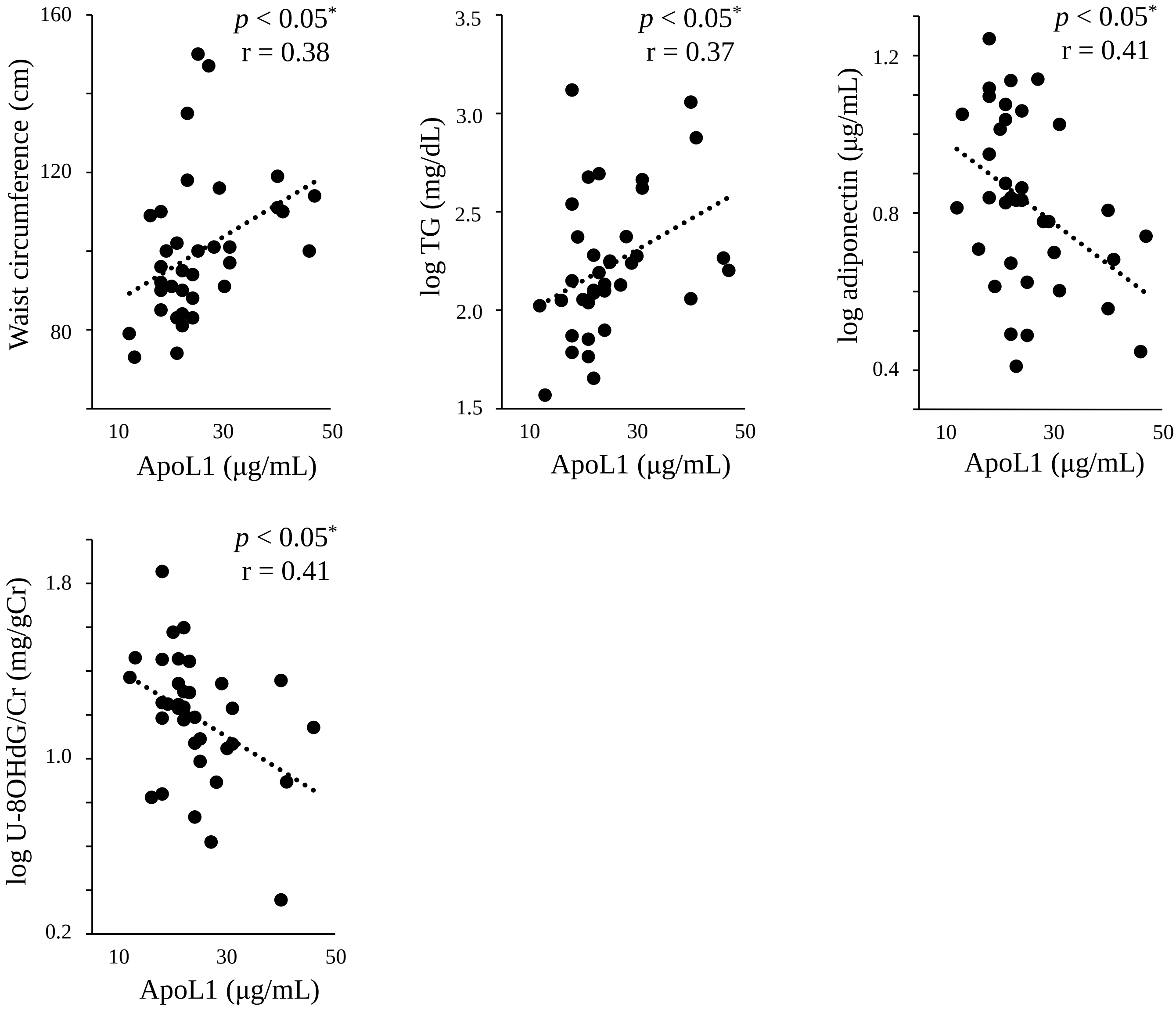

Scatter plot of serum ApoL1 levels relative to waist circumference, log TG, log adiponectin and log U-8OHdG/Cr in all patients with T2DM. ApoL1 presented the positive correlation with waist circumference and log TG, and the negative correlation with log adiponectin and log U-8OHdG/Cr. *P* value is calculated with peason’s correlation coefficient test. T2DM, type2 diabetes mellitus; TG, triglyceride; U-8OHdG/Cr, urine 8-Hydroxy-2'-Deoxyguanosine to creatinine ratio.
